# The Remote Food Photography Method and SmartIntake App for the Assessment of Alcohol Use in Young Adults: Feasibility Study and Comparison to Standard Assessment Methodology

**DOI:** 10.2196/10460

**Published:** 2018-09-24

**Authors:** Tera L Fazzino, Corby K Martin, Kelsie Forbush

**Affiliations:** 1 Department of Psychology University of Kansas Lawrence, KS United States; 2 Cofrin Logan Center for Addiction Research and Treatment University of Kansas Lawrence, KS United States; 3 Pennington Biomedical Research Center Louisiana State University System Baton Rouge, LA United States

**Keywords:** alcohol consumption, alcohol college students, alcohol assessment, dietary assessment, self report, mobile phone, mobile health, ehealth, photography, young adults

## Abstract

**Background:**

Heavy drinking is prevalent among young adults and may contribute to obesity. However, measurement tools for assessing caloric intake from alcohol are limited and rely on self-report, which is prone to bias.

**Objective:**

The purpose of our study was to conduct feasibility testing of the Remote Food Photography Method and the SmartIntake app to assess alcohol use in young adults. Aims consisted of (1) quantifying the ability of SmartIntake to capture drinking behavior, (2) assessing app usability with the Computer System Usability Questionnaire (CSUQ), (3) conducting a qualitative interview, and (4) comparing preference, usage, and alcohol use estimates (calories, grams per drinking episode) between SmartIntake and online diet recalls that participants completed for a parent study.

**Methods:**

College students (N=15) who endorsed a pattern of heavy drinking were recruited from a parent study. Participants used SmartIntake to send photographs of all alcohol and food intake over a 3-day period and then completed a follow-up interview and the CSUQ. CSUQ items range from 1-7, with lower scores indicating greater usability. Total drinking occasions were determined by adding the number of drinking occasions captured by SmartIntake plus the number of drinking occasions participants reported that they missed capturing. Usage was defined by the number of days participants provided food/beverage photos through the app, or the number of diet recalls completed.

**Results:**

SmartIntake captured 87% (13/15) of total reported drinking occasions. Participants rated the app as highly usable in the CSUQ (mean 2.28, SD 1.23). Most participants (14/15, 93%) preferred using SmartIntake versus recalls, and usage was significantly higher with SmartIntake than recalls (42/45, 93% vs 35/45, 78%; *P*=.04). Triple the number of participants submitted alcohol reports with SmartIntake compared to the recalls (SmartIntake 9/15, 60% vs recalls 3/15, 20%; *P*=.06), and 60% (9/15) of participants reported drinking during the study.

**Conclusions:**

SmartIntake was acceptable to college students who drank heavily and captured most drinking occasions. Participants had higher usage of SmartIntake compared to recalls, suggesting SmartIntake may be well suited to measuring alcohol consumption in young adults. However, 40% (6/15) did not drink during the brief testing period and, although findings are promising, a longer trial is needed.

## Introduction

Alcohol use is prevalent among young adults [1]. Most (78%) US adults aged 18-24 report drinking alcohol and 40% report heavy drinking (5+ drinks on one occasion) at least once in the previous month [1]. Heavy drinking during young adulthood is associated with a host of negative consequences, from increased risk of accidents and injuries to the development of alcohol use disorder symptoms [2]. In addition to these well-known consequences, recent evidence suggests that heavy episodic drinking during young adulthood increases the risk of excess weight gain and the transition to obesity 5 years later [3]. Drinking may disrupt energy balance directly through ingestion of calories in alcoholic beverages and indirectly through effects on alcohol-related eating [4,5]. It is important to understand the direct and indirect effects of alcohol use on energy balance and obesity risk to develop relevant obesity prevention programs.

Researchers’ ability to delineate the direct and indirect contributions of alcohol intake on energy balance, however, is limited by available measurement tools. Gold standard alcohol assessments involve asking participants to self-report the total number of drinks they consumed each day in the past 3-6 months [6]. While validity data indicate that this method may be sufficient to identify number of drinks consumed [6], it does not provide enough detail to reliably ascertain the precise caloric, nutritional, and alcoholic content of drinks. Information on the drink type, size in ounces, all alcoholic and nonalcoholic drink contents, and the amount consumed would be required to determine caloric intake from alcoholic beverages [7]. All of the aforementioned information is collected with the multiple pass 24-hour diet recall method [7]. The 24-hour diet recall method involves an iterative process through which individuals are asked to identify, for all food and beverages consumed in the past 24 hours, the food or drink type, the portion size, all contents of the food/beverage, and the amount they consumed [7]. Diet recalls have been applied to estimate caloric intake from alcohol as a component of overall energy intake in the general population [8-11]. Using data from the National Health and Nutrition Examination Survey (NHANES), researchers found that alcohol intake estimates were similar between the NHANES Alcohol Use Questionnaire, a standardized questionnaire that assesses typical quantity and frequency of alcohol use, as compared to alcohol intake estimated using diet recall data [12]. In addition, evidence suggested that 24-hour diet recalls performed similarly in measuring low to moderate levels of typical alcohol intake when compared to a 7-day retrospective recall of alcohol use, and 7-day prospectively recorded alcohol use with a food diary [13].

Despite their utility, assessments that rely on self-report are vulnerable to reporting biases due to memory inaccuracies from retrospective recall, social desirability, and inaccuracies in portion size estimates [14-16]. For example, researchers recently found that NHANES participants underestimated their intake in diet recalls by up to 800 calories per day [14], and Beasley et al [17] found that approximately 50% of the error in self-reported food intake was due to the inability of participants to accurately estimate portion size. Self-reported alcohol use suffers similar problems in underestimation [15]. A recent study of daily alcohol use found that alcoholic drink size and strength were underreported by at least 20% compared to daily alcohol use data recorded by transdermal alcohol sensors [15].

**Figure 1:**
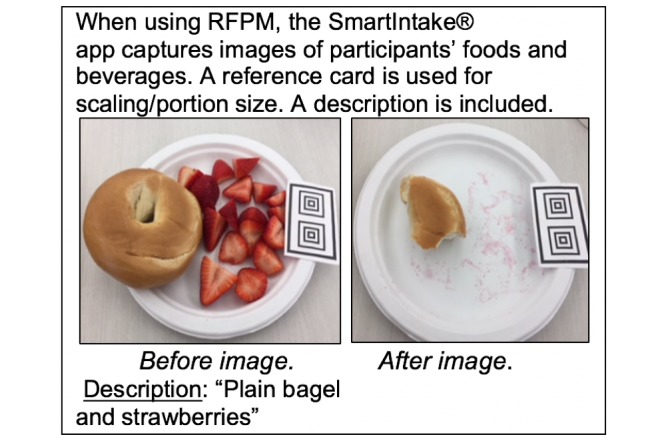
The Remote Food Photography Method (RFPM) applied using the SmartIntake app.

The Remote Food Photography Method (RFPM) was developed to address concerns regarding food and drink portion size estimation, to minimize participant burden, and to obtain accurate estimates of food and beverage intake [18-20]. With RFPM, participants capture photo images of their food selection and plate and drink waste using a mobile device in near real-time in their natural environments. Photos are analyzed by nutrition experts to estimate energy and nutrient content using standardized methods [20,21], eliminating the need for participants to accurately recall and report portion sizes. RFPM has excellent evidence for validity in measuring energy intake in the general adult population; RFPM estimates had only a 3.7% error rate when compared to energy expenditure estimates from doubly labeled water in weight-stable adults [18]. RFPM was developed prior to smartphones and has been used with various forms of mobile technology as advances have become available. RFPM was originally deployed using cellular-connected personal digital assistants, followed by camera-enabled flip phones, BlackBerry phones, and finally smartphones. For the past few years, RFPM has been deployed through a mobile phone app, SmartIntake, which can be downloaded directly onto participants’ personal mobile phones and streamlines the RFPM data collection process. Figure 1 depicts the data collection process with the RFPM and SmartIntake app.

The RFPM and SmartIntake app can be adapted to measure alcohol use in young adults to address potential inaccuracies in self-reported drink size and content. The purpose of this pilot study, therefore, was to conduct feasibility testing of the RFPM and SmartIntake app via the following four aims:

1. Quantify the ability of SmartIntake to capture drinking behavior, defined as (1) the percent of total drinking occasions captured with SmartIntake, and (2) the percent of participants who submitted alcoholic drink photos through SmartIntake. The total number of drinking occasions was determined by adding the total number of drinking occasions captured by SmartIntake plus the total number of drinking occasions participants self-reported that they failed to capture through the app.
2. Use a standard technology usability questionnaire to collect usability data for the RFPM/SmartIntake.
3. Conduct a qualitative interview to assess acceptability and feasibility of using the SmartIntake app during drinking occasions.
4. Compare preference, usage, and alcohol use estimates per drinking occasion between SmartIntake and online diet recalls, the latter of which were completed by participants for a parent study. Usage was defined by the number of days participants provided food/beverage photo data through the app, and number of diet recalls completed.

## Methods

### Ethics and Data Security

The research was approved by the Institution Review Boards at the University of Kansas and Pennington Biomedical, Louisiana State University System. All participants provided written informed consent. Due to the sensitive nature of the data collected, participants were protected under a Certificate of Confidentiality issued by the National Institutes of Health. All photos submitted through SmartIntake were not linked with participant-identifying information.

### Participants

Participants in the current study were recruited from a larger parent study. Below we first describe the parent study and then describe participant recruitment and enrollment into the current study.

### Parent Study

The parent study was designed to examine the effects of heavy alcohol use and alcohol-related eating behavior on weight gain in the first year of college. At the beginning of the academic year, interested freshmen completed an online screening that consisted of a demographics questionnaire and the Alcohol Use Disorders Identification Test–Consumption questions (AUDIT-C) to assess a pattern of heavy alcohol use [22]. A random sample of study-eligible freshmen stratified by sex (52% male), race/ethnicity (44% racial or ethnic minority), and heavy drinking status (45% endorsing a heavy drinking pattern) were enrolled (N=103).

Participants attended three study visits at the beginning, middle, and end of the 2016-2017 academic year during which they completed an alcohol assessment and provided anthropometric measurements. Following each visit, participants completed a series of three online diet recalls using the Automated Self-Administered 24-Hour Diet Recall [23], the Web-based version of the United States Department of Agriculture 5-step diet recall [24], to report their dietary intake and alcohol consumption. Diet recalls were completed on 3 days randomly selected by study staff at each assessment point—one on a weekday and two on weekend days. Participants were required to complete all diet recalls within a 1-week window and could complete recalls late if they were still within the assessment window. Participants were compensated US $15 per completed recall.

### Study Sample

The current study enrolled a convenience sample of 15 students selected from the parent study. When students attended a visit for the parent study, they were invited to participate in the current study if they endorsed a pattern of heavy drinking on the AUDIT-C at baseline or if they reported multiple (3+) recent heavy drinking episodes in the alcohol assessment. This procedure was in place to increase the likelihood that we would capture drinking episodes during the SmartIntake testing period and diet recalls. Students were also required to complete at least one diet recall (for the parent study) before starting the current study—a criterion that was met by the vast majority of participants in the parent sample. Most parent sample participants completed 1+ recall at baseline (96/103, 93%), 83% (85/103) completed 1+ recall at Visit 2, and 72% (74/103) completed 1+ recall at Visit 3. Students were consented only for the current study when their 1-week window to complete the diet recalls for the parent study had passed (to avoid overlap in assessment methods). Enrollment was conducted on a rolling basis until we reached our target (N=15).

### Procedure

**Figure 2:**
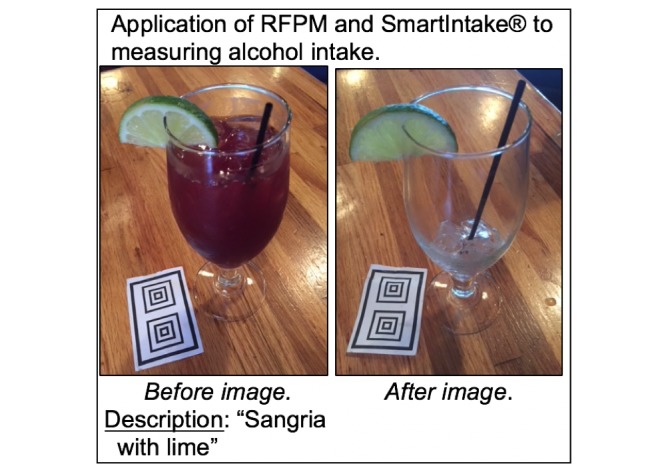
The Remote Food Photography Method (RFPM) and SmartIntake app applied to measuring alcohol intake.

Students attended an initial visit during which they provided informed consent and completed a training session to learn how to use the SmartIntake app. Participants were asked to use the app to report their food and alcohol intake for 3 consecutive days. Figure 2 depicts the RFPM and SmartIntake app process applied to alcoholic beverages.

SmartIntake testing days consisted of one weekday (Thursday) and two weekend days (Friday and Saturday). Participants returned the following week to complete a standardized app usability questionnaire and a qualitative interview about their experience using SmartIntake. Participants were not provided feedback or information about the photos they submitted (eg, alcohol calories consumed), as feedback could have altered their consumption and/or SmartIntake reporting behavior during the study.

Participants could earn up to US $60 for participating in the study. Participants were compensated US $15 per day for using SmartIntake, for a total of US $45 possible over 3 testing days. Independent of participants’ usage with app testing, participants were compensated an additional US $15 for completing the follow-up interview. The compensation structure was explained to participants during the consent process. We matched compensation for 3 days of app testing (US $15 per day; US $45 total) directly to compensation for 3 recalls (US $15 per recall; US $45 total) to facilitate comparisons between the methods.

### Measures

The Computer System Usability Questionnaire (CSUQ) is a widely used standardized questionnaire that was originally designed to measure computer program usability in field-testing studies at IBM [25,26]. The CSUQ has since been applied to studying the usability of websites [27] and mobile phone apps, including mHealth apps for adults [28-30] and adolescents [31]. This 19-item questionnaire uses a 7-point Likert scale ranging from 1 (strongly agree) to 7 (strongly disagree) and yields an overall score representing overall satisfaction with the program and three scale scores for System Usefulness, Information Quality (quality of instructions in the program and utility of error messages), and Interface Quality [32,33]. Items are averaged to obtain scores, with lower scores indicating greater usability. Evidence indicates the CSUQ has strong internal consistency across scale items and a replicable structure across tests of different types of computer programs (eg, computer, voice activated programs, Web apps) [25,26,34].

**Table 1:** Information used to calculate outcomes for Aims 1 and 4.

Dependent variables		SmartIntake app testing (3 days)	Qualitative interview	Diet recalls^a^ (3 days)
**Aim 1**				
	Percentage of drinking occasions captured by SmartIntake Percentage of participants who submitted alcohol photos	Number of total drinking occasions captured with SmartIntake Number of participants who submitted and did not submit alcohol photos	Number of total drinking occasions not captured with SmartIntake (self-reported)	N/A^b^
**Aim 4**				
	Preference	N/A	Number of participants who preferred SmartIntake and number who preferred diet recalls for reporting alcohol and food intake	N/A
	Usage	Number of days out of 3 that each participant completed SmartIntake testing	N/A	Number of recalls out of 3 that each participant completed
	Alcohol use estimates Percentage of participants who reported alcohol use	Alcohol consumption in grams and calories per drinking occasion; number of heavy drinking episodes Number of participants who submitted and did not submit alcohol photos	N/A	Alcohol consumption in grams and calories per drinking occasion; number of heavy drinking episodes Number of participants who reported and did not report alcohol use

^a^Diet recalls were completed during the parent study and used for comparisons with SmartIntake in the current study, as described in the Parent Study section of the Methods.

^b^N/A: not applicable.

The qualitative interview assessed participants’ likes and dislikes about using the app, the utility of the reminders sent from the app (these remind participants to capture images), and their experiences using SmartIntake while drinking alcohol. Participants were asked directly about instances during which they forgot or almost forgot to take photos of alcohol or food and to identify situations in which using SmartIntake might be difficult. Participants were asked to describe any circumstances during which they felt uncomfortable using the app. All questions were open-ended. Finally, participants were asked about their preference for using SmartIntake or the online diet recalls to report their alcohol and food intake.

All interviews were conducted individually with participants by the study’s principal investigator (PI). To minimize the potential for social desirability responding, the interview was framed as an opportunity for the PI to understand participants’ experiences using the app, with the purpose of working together to identify things that worked and did not work, and to hear their suggestions for improving the app and data collection methods. Participants were asked to describe times they drank alcohol and forgot to report it with the app, so that the PI could understand the circumstances under which this type of reporting did not seem feasible. Similarly, when the PI inquired about participants’ preferred method for reporting alcohol and food intake, participants were asked to explain what about the method worked best for them, so that she could understand circumstances in which one method might be preferred or work better than the other.

### Outcomes

Information used to calculate dependent variables (DV) for Aims 1 and 4 was derived from multiple sources, as detailed in Table 1. DV calculations for Aims 1-4 are presented following Table 1.

Feasibility and usability outcomes were calculated using the following metrics. For Aim 1a), the percentage of total drinking occasions captured with SmartIntake was calculated as N captured / N captured + N missed, as reported by participants. For 1b), the percentage of participants who submitted alcohol photos was calculated as N participants who submitted alcohol photos / N submitted + N who did not submit alcohol photos. For Aim 2, the CSUQ overall satisfaction score was calculated as the mean of all CSUQ items. Three scale scores for System Usefulness, Information Quality, and Interface Quality were determined by calculating the mean of items in each scale. For Aim 3, common themes were identified regarding acceptability and feasibility for using SmartIntake overall and during drinking episodes. For Aim 4, we used a repeated-measures, within-subjects design to compare preference, usage, and alcohol use estimates per drinking occasion with SmartIntake and diet recalls. Usage was defined by the number of days participants provided food/beverage photo data through the app, or number of diet recalls submitted. Alcohol use estimates per drinking occasion were calculated for SmartIntake and the diet recalls because both provide grams of alcohol consumed and caloric contents of the alcoholic beverages. Heavy drinking occasions captured through SmartIntake and the diet recalls were defined as 4+ drinks for females or 5+ for males on one occasion, in excess of low-risk drinking guidelines from the National Institute on Alcohol Abuse and Alcoholism (NIAAA) [35]. The NIAAA defines a standard drink as 14 grams of pure alcohol [35]. The total number of participants who submitted alcohol photos through SmartIntake and the total number of participants who reported alcohol use in the diet recalls were summed for comparison.

Diet recalls from the parent study that were completed at the same assessment point as SmartIntake testing were used for comparison. Because the diet recalls were completed before the current study, we did not inquire about whether participants missed reporting alcohol use in the recalls; thus, we were unable to calculate the percentage of total drinking occasions captured in diet recalls as we were for SmartIntake.

Dependent *t* tests were used to compare usage and alcohol use estimates per drinking occasion between SmartIntake and the diet recalls. Fisher exact tests were used to test the difference between number of heavy drinking episodes reported between SmartIntake and the diet recalls, and the number of participants who reported alcohol use in each method.

## Results

### Participant Characteristics

Participants (N=15) provided informed consent, tested the app, and completed the follow-up visit. Participant characteristics are presented in Table 2. On the AUDIT-C, 93% (14/15) of participants endorsed drinking alcohol 2+ times per week and one endorsed drinking 2-4 times per month. Most (13/15, 87%) reported that they engaged in weekly heavy episodic drinking.

### Aim 1: Quantifying the Ability of SmartIntake to Capture Drinking Behavior

**Figure 3:**
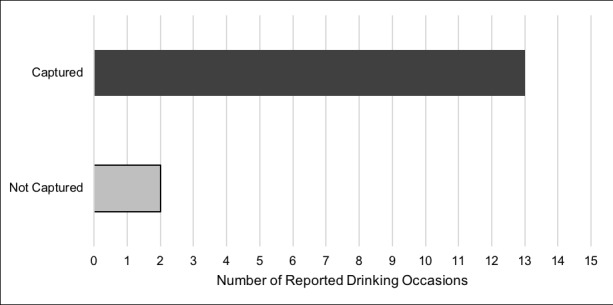
Drinking occasions captured by SmartIntake.

SmartIntake captured 87% of reported drinking occasions (Aim 1a; Figure 3). Participants submitted a total of 15 alcohol photos during 13 drinking episodes. There were two instances in which participants reported that they drank alcohol but forgot to submit photos. Both missed occasions occurred among participants who submitted other alcohol photos through SmartIntake.

Sixty percent (9/15) of participants submitted alcohol use photos through SmartIntake (Aim 1b). Of the 40% (6/15) who did not send alcohol photos through SmartIntake, all reported that they did not drink during the days they used SmartIntake.

### Aim 2: Usability

Results of the CSUQ indicated that participants were highly satisfied with SmartIntake overall (mean 2.52 on a 7-point scale, SD 1.13) and that the app was highly usable (mean 2.28, SD 1.23), provided good quality information and instructions for use (mean 2.36, SD 1.14), and had acceptable interface quality (mean 3.10, SD 1.68).

### Aim 3: Qualitative Interview to Assess Acceptability and Feasibility

#### Overall Feedback on SmartIntake

Themes from the follow-up interview largely mirrored responses to the CSUQ. Participants liked that the app was quick and easy to use and that they could report their food and beverage intake in real-time. The majority of participants indicated the reminders to submit photos were mistimed on weekends because their eating schedules were less consistent and reliable than on weekdays, despite the reminder system accommodating different schedules on the weekends. Many participants also stated they often did not notice the notifications because they were sent via email and not text message, even though the notifications showed up on their phones when their screens were locked.

**Table 2:** Participant characteristics.

Variable		Value
Age (years), mean (SD)		18.1 (0.3)
Male, n (%)		9 (60)
White, non-Hispanic, n (%)		13 (87)
**AUDIT-C score^a,b^, mean (SD)**		
	Males	7.0 (0.7)
	Females	6.3 (1.2)
Body Mass Index (BMI), mean (SD)		26.3 (6.5)
**Weight class^c^, n (%)**		
	Healthy weight	9 (60)
	Overweight	3 (20)
	Obese	3 (20)

^a^AUDIT-C: Alcohol Use Disorder Identification Test–Consumption Questions.

^b^AUDIT-C score of 5+ for females or 7+ for males indicates a pattern of heavy drinking in college students [22].

^c^Healthy weight: BMI<25 and >19; overweight: BMI=25-29.9; obese: BMI≥30.

**Table 3:** Alcohol use estimates from SmartIntake and 24-hour online diet recalls.

Alcohol estimates	SmartIntake (N=13)^a^	Diet recalls (N=7)^b^	*P* value^c^	95% CI
Alcohol grams per drinking occasion, mean (SD); range	40.0 (32.1); 11.2-95.4	40.2 (23.6); 14.0-74.9	.25	-4.03 to 15.15
Alcohol calories per drinking occasion, mean (SD)	357.0 (254.0)	375.8 (228.3)	.26	-35.43 to 128.94
Heavy drinking episodes^d^, n (%)	4 (31)	2 (29)	.99	0.10 to 16.41

^a^Alcohol reported by 60% of participants.

^b^Alcohol reported by 20% of participants.

^c^*P* values for continuous outcomes refer to within-subjects *t* tests; *P* value for count of heavy drinking episodes refers to Fisher exact test.

^d^Consumption of 4+ drinks for females, 5+ for males on one occasion, in excess of low-risk drinking guidelines from the National Institute on Alcohol Abuse and Alcoholism, which considers 14 grams of alcohol as one standard drink [35].

#### Acceptability and Feasibility of Using SmartIntake During Drinking Episodes

Most participants reported that it was feasible to take individual photos of alcoholic beverages if they were drinking with a meal. Participants reported that when they were drinking at parties or in social gatherings, it was more difficult to capture individual drink photos due to low lighting and social distractions. However, participants were trained to use the method flexibly and this appeared to facilitate data completeness. For example, during social events/parties, most participants sent summary photos of the number of drinks they consumed in one or two images. Some participants took before and after photos of liquor bottles to indicate how much they consumed. Others stacked solo cups and sent photos of all of their empty cups in one after-drinking image, along with a text description. In their interviews, participants reported that these methods helped them send data while minimizing the impact of sending photos on their social interactions.

#### SmartIntake Use in Social Situations

When asked to describe a time in which they forgot or almost forgot to take a food or drink photo, the vast majority of participants reported this happened while they were distracted in social situations and on weekends when they were not in normal routine. Both drinking occasions that participants reported they missed capturing with SmartIntake occurred in social drinking situations and were heavy drinking episodes. In addition, one third of participants (5/15) reported forgetting to submit a food photo while eating out with friends (n=4) or when eating on the run (n=1). The majority of participants (12/15, 80%) reported that using the app to record their alcohol and food intake did not make them feel uncomfortable. Three participants described feeling slightly awkward in social situations when they first started using the app due to taking out the reference card for each photo, but all reported this feeling diminished by the second or third day of app use.

### Aim 4: Within-Subjects Comparisons of SmartIntake and Diet Recalls

**Figure 4:**
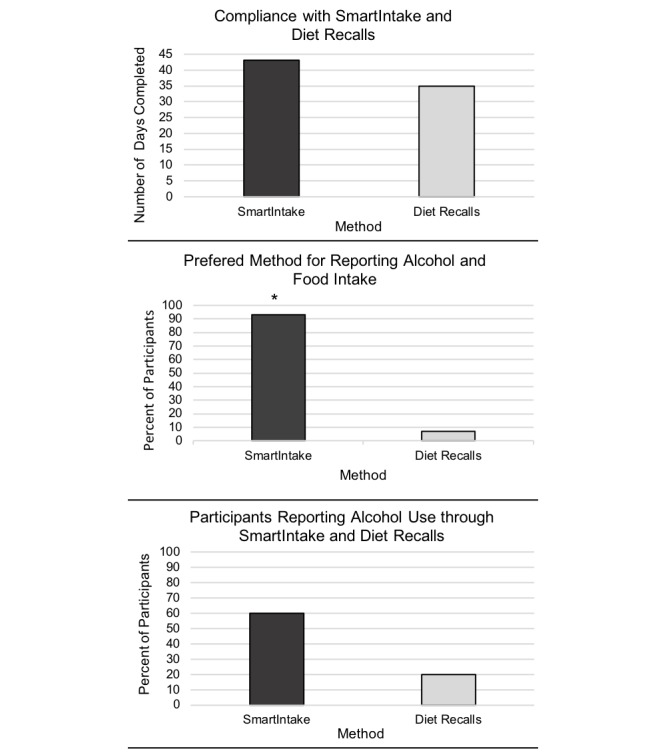
Within-subjects comparisons of SmartIntake and online diet recalls for usage, preference, and alcohol use reports. Significance test of compliance refers to within-subjects *t* test. Significance test for method preference refers to Fisher exact test. A significance of *P*<.05 is indicated by an asterisk.

Usage, preference, and alcohol use estimates are presented in Table 3 and Figure 4. Usage was significantly higher with SmartIntake versus diet recalls (*t*_14_=2.26, *P*=.04, 95% CI 0.03-1.04; Figure 2). All but one participant preferred SmartIntake over the diet recalls because it was easier to use and took less time to complete (SmartIntake, 14/15 vs diet recalls, 1/15; odds ratio [OR] 121.78; *P*<.001, 95% CI 8.67-8055.30; Figure 4). Estimates of grams and calories consumed from alcoholic drinks were not significantly different from SmartIntake estimates when alcohol was reported (Table 3). The number of participants who submitted alcohol photos using SmartIntake was triple compared to the number of participants who reported alcohol intake in the diet recalls, although the difference missed statistical significance (SmartIntake, 9/15 vs diet recalls, 3/15; OR 5.61; *P*=.06, 95% CI 0.94-44.93; Figure 4). Across all participants, total alcohol grams reported through SmartIntake was nearly double the total grams reported in recalls (SmartIntake=520.4 g vs recalls=281.3 g).

## Discussion

### Principal Considerations

The current study demonstrated that using the RFPM and SmartIntake mobile app to measure alcohol intake was feasible and well accepted by college students who endorse a pattern of heavy alcohol use. This pilot was the first to measure alcohol use via mobile photography in real-time, thus circumventing the potential for biases in participant-estimated drink size and content. Our findings indicated that SmartIntake captured the majority of reported drinking occasions. Additionally, participants preferred using SmartIntake compared to standard 24-hour diet recalls administered online due to the convenience and immediacy in submitting alcohol and food data that SmartIntake afforded. Usage with SmartIntake was significantly higher than with the diet recalls, despite the procedural advantage that existed for the diet recalls, in that they could be completed later. Alcohol use estimates per drinking occasion were similar between methods when alcohol was reported. However, the number of participants who submitted alcohol photos with SmartIntake was triple compared to the number of participants who reported alcohol use in the diet recalls. Thus, our findings suggest SmartIntake assessment may be preferable as a way to gather detailed alcohol use data from young adults.

While SmartIntake methods captured the majority of reported drinking occasions, alcohol use, and heavy drinking episodes occurred less frequently than expected, based on the drinking patterns that participants endorsed at screening. Thus, our ability to test SmartIntake for assessing a full range of drinking behavior was limited, likely in part due to our brief 3-day testing period, even though it spanned the weekend. For example, 40% of participants did not drink on the days they tested SmartIntake, although most reported typically drinking multiple times per week. In addition, while most participants endorsed a pattern of weekly heavy episodic drinking, only four drinking occasions captured through SmartIntake were heavy drinking episodes and both occasions in which participants forgot to report their alcohol use via SmartIntake were heavy drinking episodes. Thus, further work and a longer testing period is needed to comprehensively evaluate the utility of SmartIntake in assessing heavy drinking episodes and a broader range of drinking behavior.

Although SmartIntake usage was high, qualitative interviews indicated that participants did occasionally forget to send photos of alcohol and food in social situations when they were distracted. In addition, participants indicated that reminder prompts were easy to miss or disregard, even though they showed on participants’ phone screens, because they were not sent as text messages (this has been rectified in the more recent version of the SmartIntake app, version 3). However, our findings did indicate that participants found that the flexible approach to reporting alcohol use with the app was most acceptable and less disruptive in social drinking situations. Given that most drinking episodes among young adults do occur in social settings [36], our future work will be focused on further developing methods that facilitate participant response in social situations and in times of heightened distraction, while minimizing impact on their social interactions.

Mobile photo-based assessment of alcohol and food intake may be particularly well suited to young adults due to similarities with young adults’ use of mobile phones, social media, and food and drink photography. For example, the vast majority of young adults (85/103, 83%) use photo-based social media apps such as Instagram regularly [37,38], and they often use social media–based apps to display their food and beverage intake [39,40]. Further, young adults commonly use photo-based social media apps during drinking episodes, including at parties and festivals [41,42]. Thus, SmartIntake assessment may be a natural extension of young adults’ existing behavior with mobile photography of food and beverage intake. In this way, preference for and higher usage with SmartIntake as compared to diet recalls may have been influenced by participants’ greater familiarity with photographing alcohol and food intake using their smartphones.

### Strengths and Limitations

The study had several limitations. First, participants were college students and it is unclear to what degree findings would generalize to the general population or clinical populations. Second, we asked participants to self-report whether they missed capturing drinking occasions with SmartIntake, which could be subject to retrospective recall bias. However, participants attended the follow-up interview the day after they completed SmartIntake testing; thus, their memories of drinking over the past 3 days were likely sufficiently reliable for identifying number of drinks consumed [6]. We also structured the qualitative interview in a manner to limit socially desirable responding. In addition, we compared SmartIntake to diet recalls that were completed in the parent study, which resulted in all participants completing the diet recalls first, followed by SmartIntake. Thus, it is possible that the differences in alcohol report rates across the two methods may be due to other factors, such as timing in the semester. However, if semester timing did contribute to differences in alcohol use estimates, we would expect that the diet recalls would have captured more frequent alcohol reports. Drinking among college students is usually higher early and mid-semester, and lower around final exams [43]. Diet recalls were conducted earlier in the semester, while SmartIntake testing was conducted towards the end of the semester. Additionally, we did not ask participants about whether they missed reporting alcohol use in the recalls, so we do not have this information to compare directly with SmartIntake data on percentage of drinking episodes captured. Finally, our requirement that potential participants completed 1+ diet recall in the parent study may limit the generalizability of the findings to participants who did not complete recalls. However, the vast majority of participants in the parent study did complete 1+ recall at each time point (96/103, 93% at Visit 1; 85/103, 85% at Visit 2; 74/103, 72% at Visit 3); thus, our findings should generalize to the majority of the parent study sample. However, future research is needed to test the level of SmartIntake usage among individuals who do not engage with standard diet recall assessment methods.

Strengths of the study included the use of a sample that endorsed a pattern of heavy drinking, assessment of app usability via a standardized questionnaire specific to computer/app technology, and within-subjects comparison between SmartIntake and standardized assessment methodology.

### Conclusions

Photo-based mobile assessment of alcohol use with the SmartIntake app may provide a scalable, objective measure of drinking behavior that captures data in near real-time and can be remotely delivered. This methodology provides fine-grained data on caloric and nutritional content of alcoholic beverages, which will afford future opportunities to assess caloric contributions from alcohol and alcohol-related eating to weight gain and obesity in young adults. This method could also facilitate the development of future interventions that rely on real-time treatment delivery using Ecological Momentary Intervention and Just-In-Time Adaption Intervention principles.

## Abbreviations

AUDIT-C: Alcohol Use Disorder Identification Test–Consumption Questions
CSUQ: Computer System Usability Questionnaire
NHANES: National Health and Nutrition Examination Survey
NIAAA: National Institute on Alcohol Abuse and Alcoholism
RFPM: Remote Food Photography Method
